# Minimally invasive estimation of ventricular dead space volume through use of Frank-Starling curves

**DOI:** 10.1371/journal.pone.0176302

**Published:** 2017-04-27

**Authors:** Shaun Davidson, Chris Pretty, Antoine Pironet, Thomas Desaive, Nathalie Janssen, Bernard Lambermont, Philippe Morimont, J. Geoffrey Chase

**Affiliations:** 1 Department of Mechanical Engineering, University of Canterbury, Christchurch, New Zealand; 2 GIGA-Cardiovascular Sciences, University of Liège, Liège, Belgium; 3 Centre Hospitalier Universitaire de Liège, Liège, Belgium; Nagoya University, JAPAN

## Abstract

This paper develops a means of more easily and less invasively estimating ventricular dead space volume (*V*_*d*_), an important, but difficult to measure physiological parameter. *V*_*d*_ represents a subject and condition dependent portion of measured ventricular volume that is not actively participating in ventricular function. It is employed in models based on the time varying elastance concept, which see widespread use in haemodynamic studies, and may have direct diagnostic use. The proposed method involves linear extrapolation of a Frank-Starling curve (stroke volume vs end-diastolic volume) and its end-systolic equivalent (stroke volume vs end-systolic volume), developed across normal clinical procedures such as recruitment manoeuvres, to their point of intersection with the y-axis (where stroke volume is 0) to determine *V*_*d*_. To demonstrate the broad applicability of the method, it was validated across a cohort of six sedated and anaesthetised male Pietrain pigs, encompassing a variety of cardiac states from healthy baseline behaviour to circulatory failure due to septic shock induced by endotoxin infusion. Linear extrapolation of the curves was supported by strong linear correlation coefficients of *R* = 0.78 and R = 0.80 average for pre- and post- endotoxin infusion respectively, as well as good agreement between the two linearly extrapolated y-intercepts (*V*_*d*_) for each subject (no more than 7.8% variation). Method validity was further supported by the physiologically reasonable *V*_*d*_ values produced, equivalent to 44.3–53.1% and 49.3–82.6% of baseline end-systolic volume before and after endotoxin infusion respectively. This method has the potential to allow *V*_*d*_ to be estimated without a particularly demanding, specialised protocol in an experimental environment. Further, due to the common use of both mechanical ventilation and recruitment manoeuvres in intensive care, this method, subject to the availability of multi-beat echocardiography, has the potential to allow for estimation of *V*_*d*_ in a clinical environment.

## 1. Introduction

**Fig 1:**
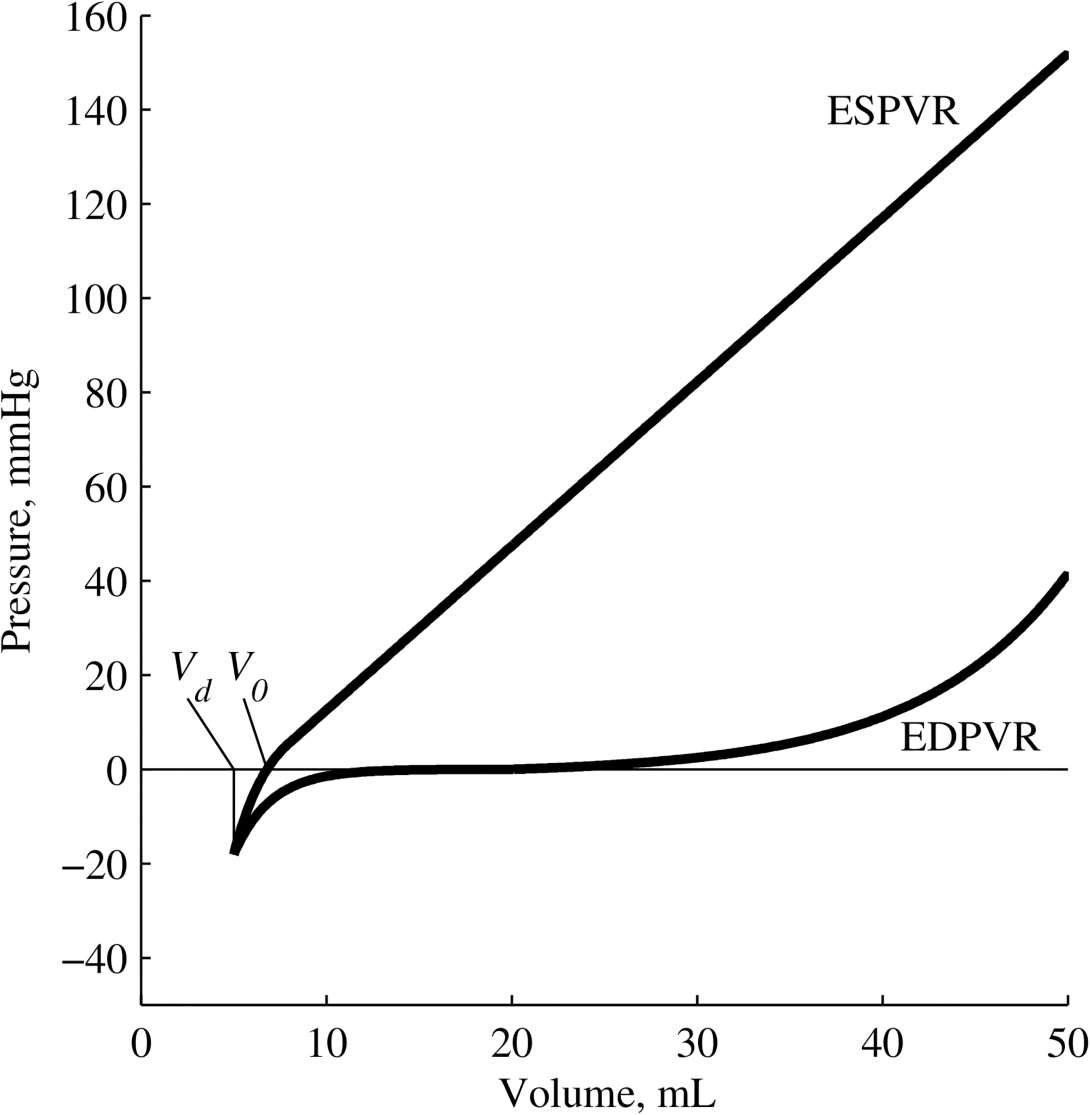
The ESPVR, *V*_*0*_ and *V*_*d*_. Definitions as in [1].

Ventricular dead space volume (*V*_*d*_) and the related ventricular volume at zero pressure (*V*_*0*_) are important subject-specific parameters for normalising inter- and intra- subject variation in cardiovascular models, including the widely used end-systolic pressure-volume relation (ESPVR) and time varying elastance (TVE) models [1–6]. *V*_*d*_ was originally conceptualised as an ‘***experimentally determined correction factor***’ for the TVE model [2] with a pair of similar physiological definitions being established. *V*_*d*_ has been said to ‘***represent a functionally dead volume at which the ventricle cannot generate any supra-atmospheric pressure***’ [1, 2], a definition generally denoted *V*_*0*_ (referred to as *V*_*0*_ henceforth). *V*_*d*_ has also been defined as the volume at which ‘***the ventricle cannot develop any systolic pressure***’, which occurs at ‘***a volume coordinate only mildly less than V***_***0***_ [1, 7]’ (referred to as *V*_*d*_ henceforth). These definitions are illustrated in Fig 1.

In cardiac models based on the TVE concept, which are widely used in haemodynamic studies [1–6], *V*_*d*_ and *V*_*0*_ are used to account for variations in heart size, shape and efficiency between individuals and as an individual’s condition changes. Specifically, *V*_*d*_ and *V*_*0*_ represent a subject and condition dependent portion of measured ventricular volume that is not actively participating in ventricular function. As such, being able to estimate and account for *V*_*d*_ and *V*_*0*_ on a subject and condition specific basis improves the physiological accuracy and the ability to accurate compare results from these models, where such models offer significant potential clinical benefits [4, 5, 8, 9]. Hence, this work presents new approaches to estimating these values to better enable the use of TVE and associated models clinically and thus enhance their clinical impact. In addition, the physical definitions of *V*_*d*_ and *V*_*0*_, and their sensitivity to contractile state, suggests a potential direct use for these terms as a diagnostic aid [6, 10]. For example, one would expect an increase in *V*_*d*_ to suggest a heart behaving less ‘efficiently’ as a pump, and, for obvious reasons, a connection between these terms and the left ventricular ejection fraction (LVEF) [11].

However, *V*_*d*_ cannot be directly measured without difficult experimental protocols due to the necessity of reducing the ventricle to atmospheric or sub-atmospheric pressure. Initial experiments validating the TVE model involved cross-circulated canine hearts [2, 3], a procedure which elegantly separates cardiac behaviour from systemic influences and allows reduction of the living heart to atmospheric pressure, but is clearly not applicable to an intensive care unit (ICU) patient. A less invasive alternative is the approximation of *V*_*0*_ (which has a similar value to *V*_*d*_) via the ESPVR [10, 12]. However, this procedure typically relies on occlusion of the vena cava, a specialised intervention that places a significant added burden on both medical staff and patients, and thus is largely constrained to experimental studies. Further, the short time interval necessitated by such a procedure means that transient, rather than steady state, behaviour is captured unless cardiac reflexes are suppressed, obfuscating the true ESPVR curve [13, 14]. As such, there is no easy, practical means to assess *V*_*d*_ or *V*_*0*_ in the ICU, where it might add clinical value.

This paper presents a novel method for deriving *V*_*d*_ as physiologically defined in [7]: the volume at which ‘the ventricle cannot develop any systolic pressure’. It relies upon the extrapolation of a Frank-Starling curve (*SV-V*_*ed*_) and its end-systolic equivalent (*SV-V*_*es*_) to the point where stroke volume (*SV*) is 0, and ‘the ventricle cannot develop any systolic pressure’. The method utilises common ICU procedures, such as mechanical ventilation recruitment manoeuvres, to develop this curve, and does not require specialised clinical intervention. The method is demonstrated across both a healthy, baseline case as well as a compromised state after an endotoxin infusion to demonstrate the method’s applicability to both healthy and compromised cardiovascular systems.

The method as presented here employs an invasive left ventricular catheter to measure end-diastolic (*V*_*ed*_) and end-systolic (*V*_*es*_) volume. Such volume measurements are increasingly available non-invasively via methods such as echocardiography [15], though it is important to note that the number of *V*_*ed*_ and *V*_*es*_ measurements required are demanding by the standards of modern echocardiography. While further validation and a modified protocol would be required, this method has the long-term potential to allow non-additionally invasive, patient-specific evaluation of *V*_*d*_ in a clinical environment, and thus an assessment of its clinical value beyond use in physiological models.

## 2. Materials and methods

### 2.1 Ethical approval

All experimental procedures and protocols used in this investigation were reviewed and approved by the Institutional Animal Care and Use Ethics Committee of the University of Liège, Belgium (Reference Number 14–1726). Their guidelines conform completely with the *Guide for the Care and Use of Laboratory Animals* published by the US National Institutes of Health (NIH Publication No. 85–23, revised 1996), as well as *EU DIRECTIVE 2010/63/EU* on the protection of animals used for scientific purposes.

### 2.2 Experimental procedure

Five male, pure Pietrain pigs weighing between 18.5 and 29 kg were sedated and anesthetised by an initial intramuscular dose of Zoletil 100 (0.1 mL/kg) and Ketamine 1000 (0.1 mL/kg). Sedation and anaesthesia was maintained by a continuous infusion of Nimbex (1 mL/kg/h at 2 mg/mL), Sufenta (0.1 mL/kg/h at 0.005 mg/mL) and Thiobarbital (0.1 mL/kg/h) via a central venous catheter positioned within the superior vena cava. The pigs were mechanically ventilated (GE Engstrom CareStation) with a baseline positive end-expiratory pressure (PEEP) of 5 cmH_2_O and tidal volume of 270 mL. The heart was accessed via a median sternotomy, and an admittance pressure-volume catheter (Transonic, NY, USA) with a sampling rate of 250 Hz inserted into the left ventricle. Proximal aortic pressure was continually sampled using a pressure catheter (Transonic, NY, USA) with a sampling rate of 250 Hz. Euthanasia was performed via a bolus of Pentobarbital (30 mg/kg) and Sufentanil (5 μg/kg) causing respiratory arrest.

To ensure a diverse range of cardiac states was exhibited, several procedures were performed:

- A single infusion of endotoxin (lipopolysaccharide from E. Coli, 0.5 mg/kg injected over 30 minutes) to induce septic shock, which drives a change in afterload conditions and is associated with a large variety of effects including an inflammatory response and capillary leakage that may lead to hypovolemia, global tissue hypoxia and cardiac failure [16].
- Several PEEP driven recruitment manoeuvres (both pre- and post- endotoxin infusion), which drive a change in preload conditions and are typically associated with a decrease in mean blood pressure and cardiac output [17].
- One to four infusions of 500 mL saline solution over 30 minute periods (both pre- and post- endotoxin infusion), simulating fluid resuscitation therapy, a key component of hemodynamic resuscitation in patients with severe sepsis, which results in a change in circulatory volume [18].

### 2.3 Estimation of *V*_*d*_

This method uses the definition of *V*_*d*_ as the volume at which ‘the ventricle cannot develop any systolic pressure’ [1, 7]. Left ventricular end-systolic (*V*_*es*_), end-diastolic (*V*_*ed*_) and stroke (*SV*) volumes for each heartbeat were established from catheter data. This data was used to generate a Frank-Starling curve (*SV*-*V*_*ed*_) and its end-systolic equivalent (*SV-V*_*es*_) for each subject. Separate linear regression of the *SV-V*_*es*_ and *SV-V*_*ed*_ curves was performed using a total least squares algorithm [19]. Independent extrapolation of each curve to the y-axis should result in both converging to a single point at which *SV* = 0. At this point, no systolic pressure is developed over the course of a heartbeat, as *V*_*ed*_ = *V*_*es*_, and *V*_*d*_ is the value of the ventricular volume y-intercept.

### 2.4 Evaluation of method

Ventricular Dead Space Volume (*V*_*d*_) is extremely difficult to physically measure, and some ambiguity as to the exact definition of this value exists [1]. These difficulties make validation via a measured ‘ground truth’ *V*_*d*_ value impractical. As such, validation of the method used to derive *V*_*d*_ must rely on validating individual model assumptions and the physiological reasonability of the results. This process encompasses:

- Evaluation of the agreement between the separate *V*_*d*_ values derived from the *SV-V*_*es*_ and *SV-V*_*ed*_ curves for each pig. Theoretically, the two curves should intersect at an identical *V*_*d*_ value if the linear approximation of the Frank-Starling curve holds.
- Assessment of the validity and strength of linear regression via Pearson’s correlation coefficients (presented as R values) to ensure a linear, physiological relationship, rather than chance, creates the observed lines.
- Evaluation of the reasonability of the derived *V*_*d*_ values, both in terms of physiology and compared to values presented in literature.

The analysis of data for each pig was separated into pre- and post- endotoxin infusion, as the development of sepsis should modify contractility and result in an increase in *V*_*d*_. This hypothesis was evaluated using a one-tailed paired Wilcoxon Signed-Rank Test [20]. This non-parametric statistical test does not rely on data being normally distributed. A single value for *V*_*d*_ pre- and post- endotoxin infusion was provided from each of 6 pigs (*n* = 6). Overall, the method was employed across a total of 59,513 heartbeats worth of data, and 6 different animals in multiple circulatory states.

**Fig 2:**
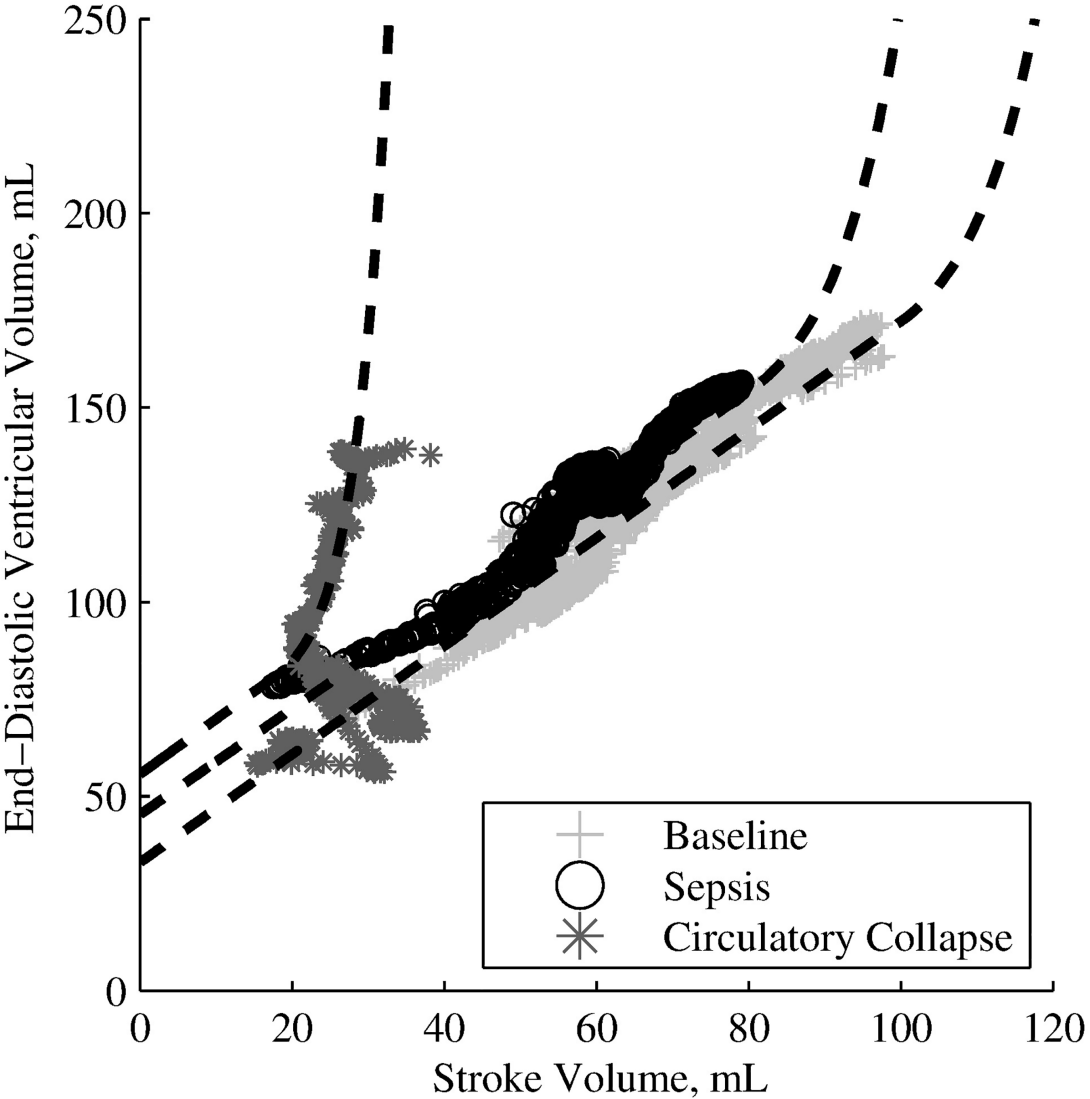
Example Frank-Starling curves with data from Pig 3 overlaid. 6841 heartbeats, illustrative trend lines drawn by hand.

Once severe sepsis developed, the Frank-Starling curve generally collapsed to the extent nonlinear behaviour was present in the observable data range. Data gathered during this period of severe sepsis, the onset of which was defined as a drop in LVEF of greater than 33% for greater than 60 seconds [21], is excluded, as linear behaviour is a poor approximation to make at this point. This observable non-linear behaviour is illustrated by the Frank-Starling curves (approximated by hand) and the ‘circulatory collapse’ region in Fig 2, overlaid with data from Pig 3.

## 3. Results

Table 1 shows the absolute and relative values of *V*_*d*_ derived from the end-systolic (*V*_*d*_(*V*_*es*_)) and end-diastolic (*V*_*d*_(*V*_*ed*_)) curves, as well as the variation between these values. *V*_*d*_ values for each subject are specified separately for the pre- and post-endotoxin infusion periods. Due to the relatively small number of subjects, it is possible the data set is not normally distributed and thus overall values are presented as median (25^th^ percentile–75^th^ percentile).

**Table 1:** *V*_*d*_ and its variability, as determined by linear regression.

Pig		*V*_*d*_(*V*_*es*_)^a^, mL	*V*_*d*_(*V*_*ed*_)^a^, mL	Δ*V*_*d*_, mL	Δ*V*_*d*_, %*V*_*d*_(*V*_*es*_)	*V*_*es*_(*Bas*)^b^, mL	*V*_*d*_(*V*_*es*_), %*V*_*es*_(*Bas*)	*V*_*d*_(*V*_*ed*_), %*V*_*es*_(*Bas*)
Pig 1	N^c^	26.8	27.5	0.7	2.7%	60.6	44.3%	45.5%
	S^c^	33.6	34.0	0.4	1.2%		55.5%	56.2%
Pig 2	N	31.9	31.2	0.7	2.2%	71.2	44.7%	43.8%
	S	35.1	35.4	0.3	0.9%		49.3%	50.0%
Pig 3	N	22.8	22.8	0.1	0.4%	49.3	46.2%	46.4%
	S	40.7	40.2	0.5	1.2%		82.6%	81.5%
Pig 4	N	29.8	32.1	2.3	7.8%	61.6	48.3%	52.0%
	S	43.2	43.8	0.6	1.4%		70.1%	71.0%
Pig 5	N	26.2	26.8	0.6	2.3%	52.6	49.8%	50.9%
	S	41.8	42.0	0.2	0.4%		79.4%	79.7%
Pig 6	N	27.1	26.0	1.1	4.1%	51.1	53.1%	50.9%
	S	32.3	32.2	0.1	0.4%		63.2%	62.9%
Overall ^d^	N	27.0 (26.2–29.8)	27.2 (26.0–31.2)	0.7 (0.6–1.1)	2.5% (2.2–4.1)	57.7	47.3% (44.7–49.8)	48.7% (45.5–50.9)
	S	37.9 (33.6–41.8)	37.8 (34.0–42.0)	0.4 (0.2–0.5)	1.1% (0.4–1.2)		66.7% (55.5–79.4)	67.0% (56.2–79.7)

^a^ *V*_*d*_(*V*_*es*_) and *V*_*d*_(*V*_*ed*_) denote *V*_*d*_ values derived from the *SV*-*V*_*es*_ and *SV*-*V*_*ed*_ curves respectively.

^b^ *V*_*es*_(*Bas*) denotes the baseline end-systolic volume, averaged over the first 10 heartbeats of the experiment.

^c^ N denotes data from the pre-infusion (normal) region while S denotes data from the post-infusion (developing sepsis) region.

^d^ Overall values are presented as median (25^th^ percentile–75^th^ percentile).

The method presented relies on the assumption that stroke volume is linearly correlated to *V*_*es*_ and *V*_*ed*_, and that linear extrapolation of this relationship is valid. These assumptions are supported by the strong agreement between the two separately established values of *V*_*d*_, with an overall median absolute difference Δ*V*_*d*_ of 2.5% pre- endotoxin infusion and 1.1% post- endotoxin infusion, for each pig. The values of *V*_*d*_ are also physiologically reasonable and consistent at 44.3–53.1% of baseline *V*_*es*_ pre- endotoxin infusion [10] and 49.3–82.6% of baseline *V*_*es*_ post- endotoxin infusion for all pigs. Further, the overall values for *V*_*d*_ rose significantly (*p* = 0.0156, one-tailed paired Wilcoxon Signed-Rank Test [20]) as sepsis developed, thus showing the impact of sepsis in reducing cardiac function using this measure. However, the small sample size (*n* = 6) should be noted.

**Fig 3:**
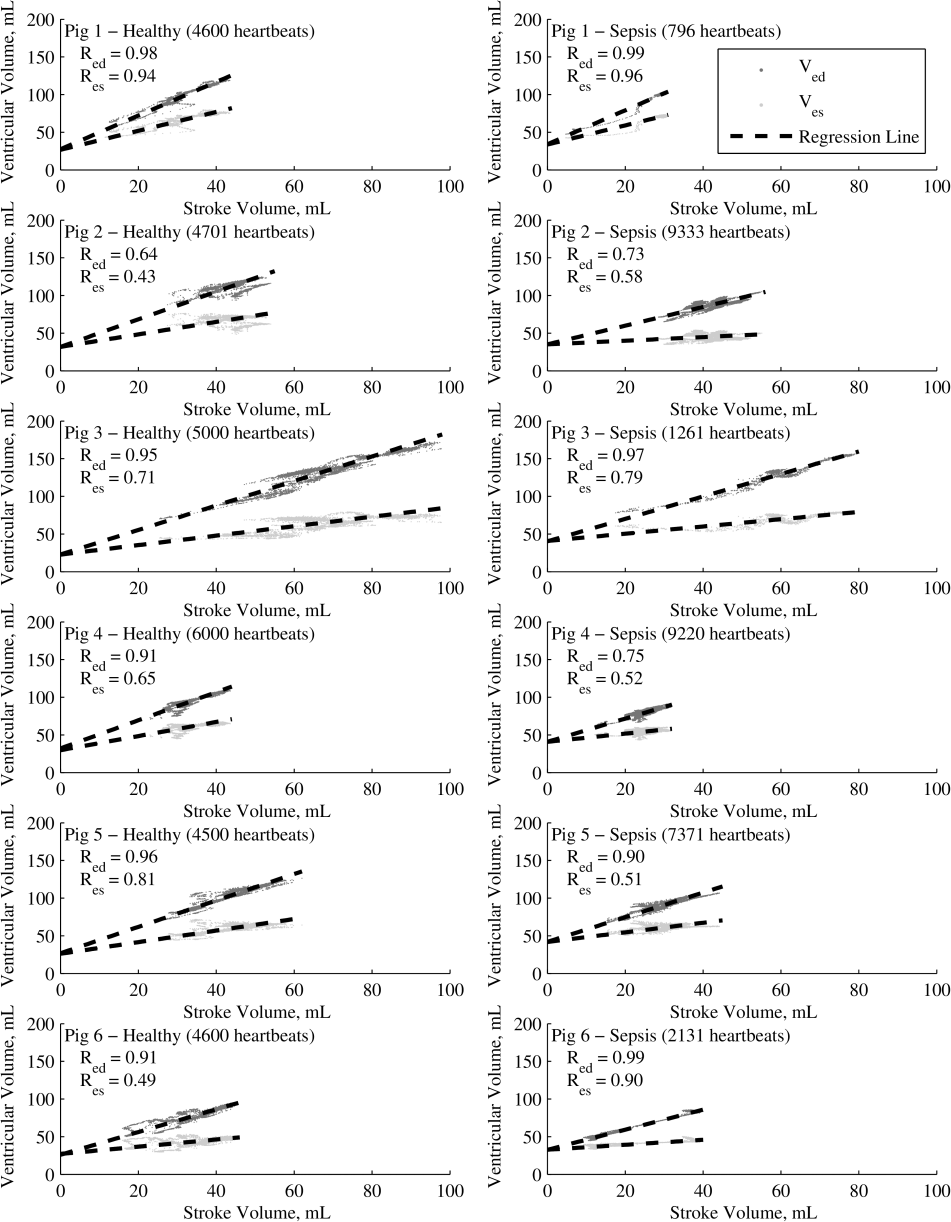
Pre- (left) and post- (right) endotoxin infusion *SV*-*V*_*ed*_, *SV*-*V*_*es*_ curves and regression lines for each pig. Number of heartbeats and Pearson’s Correlation Coefficients (*R*) presented in figure.

Fig 3 shows the *SV*-*V*_*es*_ and *SV*-*V*_*ed*_ curves used to derive *V*_*d*_, and the corresponding regression lines and correlation coefficients for each pig, pre- and post- endotoxin infusion. The stroke and absolute volumes for each pig occupy a reasonably diverse range both inter- and intra- subject. The correlation coefficients, *R* = 0.78 and *R* = 0.80 average for pre- and post- endotoxin infusion respectively, are consistently high, again supporting the validity of a linear model over these periods and interventions. The lower correlation coefficients for *SV-V*_*es*_ are primarily due to the lower gradients present in this curve, which is close to horizontal for several pigs, as observed in this work and others [22].

## 4. Discussion

### 4.1 Validation of method

One of the core assumptions made in determining *V*_*d*_ is that stroke volume (*SV*) is a linear function of both end-systolic (*V*_*es*_) and end-diastolic (*V*_*ed*_) ventricular volumes. This assumption is somewhat physiologically intuitive, as both *V*_*es*_ and *V*_*ed*_ are intrinsically related and largely reliant on the same underlying factors, and such behaviour has been observed elsewhere [23]. However, one would expect some degree of independent variation, especially as sepsis and thus circulatory distress progresses. Fig 3 shows consistently strong linear relationships for *SV-V*_*es*_ (*R* = 0.69 average) and *SV*- *V*_*ed*_ (*R* = 0.89 average) for all 6 pigs undergoing a full progression from healthy to cardiac failure due to sepsis. This result implies that, while some independent variation in *V*_*es*_ and *V*_*ed*_ certainly occurs, the variables largely maintain a linear relationship even when the cardiac system is under considerable stress.

Another core assumption is that the relationship between *SV*, *V*_*es*_ and *V*_*ed*_ is not just linear in the observed range, but can be linearly extrapolated to *SV* = 0. Table 1 and Fig 3 both show that independent linear extrapolation of the *SV-V*_*es*_ and *SV*-*V*_*ed*_ lines for each pig yields extremely consistent values for *V*_*d*_ with 7.8% variation at most, and an overall median absolute difference in paired *V*_*d*_ estimates of 2.1%. This low degree of variation across 6 separate subjects exhibiting a range of cardiac behaviour across a diverse and demanding clinical protocol provides strong support for this assumption. Further, the Frank-Starling Curve (*SV-V*_*ed*_) is known to behave linearly in this region [24], and linear behaviour of the *SV-V*_*es*_ curve may thus also be reasonably intuited.

Further supporting evidence of the validity of linear extrapolation is provided in the fact that the method yields extremely consistent, positive, values for *V*_*d*_ relative to *V*_*es*_. These *V*_*d*_ values are 44.3–53.1% of the baseline *V*_*es*_ pre-infusion, and 49.3–82.6% of the baseline *V*_*es*_ post-infusion. This consistency supports the idea that *V*_*d*_ varies between subjects in a manner somewhat proportional to ventricular volume for a given set cardiac conditions. The fact that this percentage range of baseline *V*_*es*_ is reasonably small for healthy behaviour provides the possibility to estimate baseline *V*_*d*_ from baseline *V*_*es*_, though this range of values may be different for humans compared to pigs.

The derived values for *V*_*d*_ also agree with measured values provided in the literature. Although these typically use the *V*_*d*_ = *V*_*0*_ definition for ventricular dead space, the two values have been shown to be similar [1, 7]. For example [10] presents several *V*_*0*_ values for cross circulated dogs, and protocols involving preload, afterload and inotropic variation. The baseline preload experiment gives a range of 13–52% and the baseline afterload experiment a range of 46–83% of *V*_*es*_ for *V*_*0*_, overlapping with the 44.3–53.1% of *V*_*es*_ range for *V*_*d*_ observed in this paper. Observations in [6], a study conducted on humans, yield *V*_*0*_ values for a normal contractile state in a 24–84% of *V*_*es*_ range for *V*_*0*_ (ignoring negative results) across 5 subjects, again encompassing the 44.3–53.1% of *V*_*es*_ range for *V*_*d*_ observed in this paper. Further, [6] also observed an increase in *V*_*0*_ values from a baseline average value of 32 cc/m^2^ to an average value of 46 cc/m^2^ for individuals with intermediate and 100 cc/m^2^ for individuals with poor contractile function, which agrees with the statistically significant increase in *V*_*d*_ observed in this paper as sepsis developed. Other values presented in literature are similarly sized, positive values, as might be expected [12, 25]. Overall, this further data provides a strong body of evidence for the physiological validity of the results and thus the proposed methodology.

### 4.2 Response to sepsis

In the period between endotoxin infusion and cardiac failure, *V*_*d*_ values rose in all cases from a range of 44.3–53.1% to 49.3–82.6%. This statistically significant increase in dead space volume corresponds to the decreased LVEF observed in sepsis patients, and the myocardial depression associated with failure to survive sepsis [26]. This behaviour agrees well with the physiological definition of *V*_*d*_, and supports its potential clinical use as a diagnostic aid. Importantly, in this region, the agreement between the two *V*_*d*_ values remained strong, and correlation coefficients remained high, suggesting the Frank-Starling curve remained in its linear region despite significant change in cardiac function.

Directly prior to death, the Frank-Starling curve generally underwent a dramatic shift to the left and nonlinear behaviour was observable and strong (example in Fig 2). This corresponds to the expected behaviour of the Frank-Starling curve as complete cardiac failure occurs [27]. This data, characterised by a decrease in LVEF of greater than 33% for longer than 60 seconds, was excluded from the ‘post-endotoxin infusion’ data as linear behaviour is a poor approximation to make at this point.

### 4.3 Limitations

There are some limitations to this study worth discussing. First, the study employs a left ventricular catheter to measure *V*_*ed*_ and *V*_*es*_, the insertion of which is an invasive procedure that is not common practice in the ICU. Such volume measurements are increasingly available non-invasively via methods such as echocardiography [15], but the number of measurements required for this method is demanding by current echocardiography standards. Further, volume measurement, in addition to specialised invasive protocols, is also required for approximation of *V*_*0*_ via the ESPVR [13, 14], which is the standard method available for finding a value similar to *V*_*d*_. In contrast, the method presented here requires only common ICU interventions, primarily recruitment manoeuvres, to obtain a large enough range of data to fit a line. These interventions occur in normal care and are thus less burdensome to obtain. They also occur over a longer timescale meaning cardiac reflexes distort results less significantly compared to other methods.

Additionally, all data presented here is the result of the same protocol involving sepsis, a complex and varied condition [16], and several standardised interventions. While this data set encompasses several pigs and the full progression from healthy, baseline behaviour to cardiac failure, there are a huge range of possible cardiac conditions that could be tested. Thus further validation over several of these conditions would be beneficial. Regardless, the underlying physiology and data supporting the development of this method has been discussed in detail, and would be expected to transfer to the majority of such conditions.

### 4.4 Summary

Overall, *V*_*d*_ is an important, subject specific, physiological value that is difficult to measure or accurately approximate, even when invasive instrumentation is available [1]. Thus, this method offers significant potential in its ability to provide a relatively easy, non-additionally invasive means of estimating *V*_*d*_ when ventricular volume measurements are available, without requiring a specific and highly involved protocol. Though any clinical application will require further studies and a modified protocol, this method offers the potential to aid in assessment of patient condition through its ability to normalise intra- and inter- patient variability.

## 5. Conclusion

Ventricular dead space volume (*V*_*d*_) is an important, subject specific value for normalisation of inter- and intra-subject variation. However, its definition is ambiguous and it is difficult to directly measure or approximate in a clinical environment. A method is presented involving linear extrapolation of a Frank-Starling curve and its end-systolic equivalent, which allows subject specific estimation of *V*_*d*_, while only requiring typical ICU procedures. The method yielded good agreement in *V*_*d*_ values (7.8% variation at most), is based on strong linear correlations (R = 0.79 average) and produced physiologically reasonable values for *V*_*d*_ (44.3–53.1% and 49.3–82.6% of baseline *V*_*es*_ before and after endotoxin infusion respectively). Overall, this method has the potential in the longer term to allow estimation of *V*_*d*_ and thus an increased ability to normalise inter-subject variation in a clinical environment.

## References

[1] Sagawa K. Editorial: the end-systolic pressure-volume relation of the ventricle: definition, modifications and clinical use. Circulation. 1981;63(6). 10.1161/01.cir.63.6.1223 7014027

[2] Suga H, Sagawa K, Shoukas AA. Load independence of the instantaneous pressure-volume ratio of the canine left ventricle and effects of epinephrine and heart rate on the ratio. Circ Res. 1973;32(3):314–22. 4691336 10.1161/01.res.32.3.314

[3] Suga H, Sagawa K. Instantaneous pressure-volume relationships and their ratio in the excised, supported canine left ventricle. Circ Res. 1974;35(1):117–26. 4841253 10.1161/01.res.35.1.117

[4] Stevenson D, Revie J, Chase JG, Hann CE, Shaw GM, Lambermont B, et al Algorithmic processing of pressure waveforms to facilitate estimation of cardiac elastance. Biomed Eng Online. 2012;11(1):1–16. 22703604 10.1186/1475-925X-11-28 PMC3533753

[5] Stevenson D, Revie J, Chase JG, Hann CE, Shaw GM, Lambermont B, et al Beat-to-beat estimation of the continuous left and right cardiac elastance from metrics commonly available in clinical settings. Biomed Eng Online. 2012;11:73 doi: 10.1186/1475-925X-11-73 22998792 10.1186/1475-925X-11-73 PMC3538613

[6] Grossman W, Braunwald E, Mann T, McLaurin L, Green L. Contractile state of the left ventricle in man as evaluated from end-systolic pressure-volume relations. Circulation. 1977;56(5):845–52. 71960 10.1161/01.cir.56.5.845

[7] Sunagawa K, Maughan W, Friesinger G, Chang M, Sagawa K, editors. Coronary Perfusion-Pressure and Left-Ventricular Endsystolic Pressure-Volume Relation. Circulation; 1980: American Heart Association.

[8] Pironet AD, T.; Chase, J. G.; Morimont, P.; Dauby, P. C. Model-Based Computation of Total Stressed Blood Volume from a Preload Reduction Experiment. 2013. 10.1016/j.mbs.2015.03.015 25865932

[9] Kamoi S, Pretty C, Docherty P, Squire D, Revie J, Chiew YS, et al Continuous stroke volume estimation from aortic pressure using zero dimensional cardiovascular model: proof of concept study from porcine experiments. PLoS One. 2014;9(7):e102476 doi: 10.1371/journal.pone.0102476 25033442 10.1371/journal.pone.0102476 PMC4102500

[10] Kass D, Maughan W, Guo ZM, Kono A, Sunagawa K, Sagawa K. Comparative influence of load versus inotropic states on indexes of ventricular contractility: experimental and theoretical analysis based on pressure-volume relationships. Circulation. 1987;76(6):1422–36. 3454658 10.1161/01.cir.76.6.1422

[11] Paulus WJ, Tschöpe C, Sanderson JE, Rusconi C, Flachskampf FA, Rademakers FE, et al How to diagnose diastolic heart failure: a consensus statement on the diagnosis of heart failure with normal left ventricular ejection fraction by the Heart Failure and Echocardiography Associations of the European Society of Cardiology. Eur Heart J. 2007. 10.1093/eurheartj/ehm037 17428822

[12] Mehmel H, Stockins B, Ruffmann K, Von Olshausen K, Schuler G, Kübler W. The linearity of the end-systolic pressure-volume relationship in man and its sensitivity for assessment of left ventricular function. Circulation. 1981;63(6):1216–22. 7226470 10.1161/01.cir.63.6.1216

[13] Van der Velde E, Burkhoff D, Steendijk P, Karsdon J, Sagawa K, Baan J. Nonlinearity and load sensitivity of end-systolic pressure-volume relation of canine left ventricle in vivo. Circulation. 1991;83(1):315–27. 1670628 10.1161/01.cir.83.1.315

[14] Sato T, Shishido T, Kawada T, Miyano H, Miyashita H, Inagaki M, et al ESPVR of in situ rat left ventricle shows contractility-dependent curvilinearity. Am J Physiol Heart Circ Physiol. 1998;274(5):H1429–H34. 10.1152/ajpheart.1998.274.5.H1429 9612346

[15] Kirkpatrick E, Shillingford AJ, Cohen MS. Echocardiography in the ICU. Pediatric and Congenital Cardiology, Cardiac Surgery and Intensive Care: Springer; 2014 p. 879–99.

[16] Nguyen HB, Rivers EP, Abrahamian FM, Moran GJ, Abraham E, Trzeciak S, et al Severe sepsis and septic shock: review of the literature and emergency department management guidelines. Ann Emerg Med. 2006;48(1):54. e1. 10.1016/j.annemergmed.2006.02.015 16781920

[17] Jardin F, Farcot J-C, Boisante L, Curien N, Margairaz A, Bourdarias J-P. Influence of positive end-expiratory pressure on left ventricular performance. N Engl J Med. 1981;304(7):387–92. doi: 10.1056/NEJM198102123040703 7005679 10.1056/NEJM198102123040703

[18] Vincent J-L, Gerlach H. Fluid resuscitation in severe sepsis and septic shock: an evidence-based review. Crit Care Med. 2004;32(11):S451–S4. 15542955 10.1097/01.ccm.0000142984.44321.a4

[19] Golub GH, Van Loan C. Total least squares. Smoothing Techniques for Curve Estimation: Springer; 1979 p. 69–76.

[20] Randles RH. Wilcoxon signed rank test. Encyclopedia of statistical sciences. 1988.

[21] Hunter J, Doddi M. Sepsis and the Heart. Br J Anaesth. 2010;104(1):3–11. doi: 10.1093/bja/aep339 19939836 10.1093/bja/aep339

[22] Lee Rodgers J, Nicewander WA. Thirteen ways to look at the correlation coefficient. Am Stat. 1988;42(1):59–66.

[23] Faes TJ, Kerkhof PL. The Volume Regulation Graph versus the Ejection Fraction as Metrics of Left Ventricular Performance in Heart Failure with and without a Preserved Ejection Fraction: A Mathematical Model Study. Clin Med Insights Cardiol. 2015;9(Suppl 1):73 doi: 10.4137/CMC.S18748 26052232 10.4137/CMC.S18748 PMC4446890

[24] Glower DD, Spratt JA, Snow ND, Kabas J, Davis J, Olsen C, et al Linearity of the Frank-Starling relationship in the intact heart: the concept of preload recruitable stroke work. Circulation. 1985;71(5):994–1009. 3986986 10.1161/01.cir.71.5.994

[25] Davidson S, Kannangara, DO, Pretty, CG, Kamoi, S, Pironet, A, Desaive, T, Chase, JG, editor Modelling of the Nonlinear End-Systolic Pressure-Volume Relation and Volume-at-Zero-Pressure in Porcine Experiments. Conf Proc IEEE Eng Med Biol Soc; 2015 August 25–29, 2015; Milan, Italy. 10.1109/EMBC.2015.7319892 26737792

[26] Fernandes CJ Jr, de Assuncao MSC. Myocardial dysfunction in sepsis: a large, unsolved puzzle. Crit Care Res Pract. 2012;2012. 10.1155/2012/896430 PMC3312225 22482045

[27] Sarnoff SJ, Berglund E. Ventricular function I. Starling's law of the heart studied by means of simultaneous right and left ventricular function curves in the dog. Circulation. 1954;9(5):706–18. 13161102 10.1161/01.cir.9.5.706

